# Ex Pluribus Unum: The CD4 T Cell Response against Influenza A Virus

**DOI:** 10.3390/cells13070639

**Published:** 2024-04-05

**Authors:** Caroline M. Finn, K. Kai McKinstry

**Affiliations:** Immunity and Pathogenesis Division, Burnett School of Biomedical Sciences, College of Medicine, University of Central Florida, Orlando, FL 32827, USA; caroline.finn@ucf.edu

**Keywords:** CD4 T cell, influenza virus, Th1, Th17, follicular helper cell, regulatory CD4 T cell, cytotoxic CD4 T cell

## Abstract

Current Influenza A virus (IAV) vaccines, which primarily aim to generate neutralizing antibodies against the major surface proteins of specific IAV strains predicted to circulate during the annual ‘flu’ season, are suboptimal and are characterized by relatively low annual vaccine efficacy. One approach to improve protection is for vaccines to also target the priming of virus-specific T cells that can protect against IAV even in the absence of preexisting neutralizing antibodies. CD4 T cells represent a particularly attractive target as they help to promote responses by other innate and adaptive lymphocyte populations and can also directly mediate potent effector functions. Studies in murine models of IAV infection have been instrumental in moving this goal forward. Here, we will review these findings, focusing on distinct subsets of CD4 T cell effectors that have been shown to impact outcomes. This body of work suggests that a major challenge for next-generation vaccines will be to prime a CD4 T cell population with the same spectrum of functional diversity generated by IAV infection. This goal is encapsulated well by the motto ‘*ex pluribus unum*’: that an optimal CD4 T cell response comprises many individual specialized subsets responding together.

## 1. Introduction

**Figure 1:**
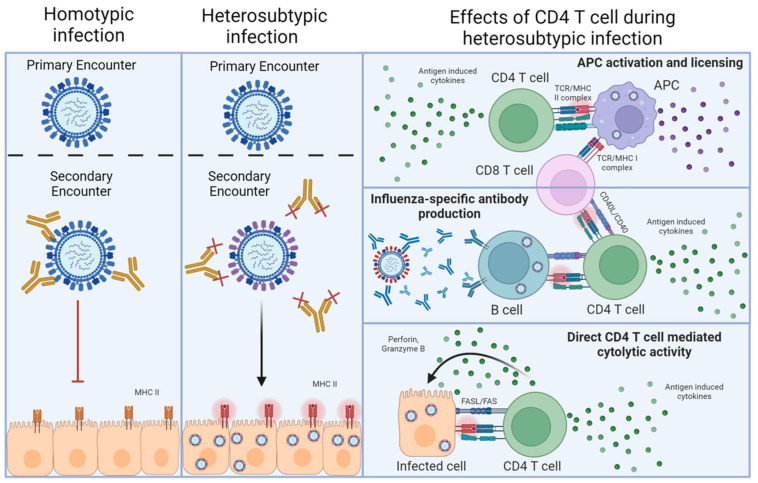
Homotypic versus heterosubtypic immunity against IAV. The left-hand column depicts homotypic re-infection of an IAV-primed individual. In this case, neutralizing antibodies against the HA and NA of the challenge virus (or vaccine) preexist, which can prevent infection upon reencounter with a virus expressing the same HA and NA. The middle column depicts heterosubtypic re-infection, where prexisting antibodies raised by an initial IAV encounter cannot neutralize the newly encountered IAV, but T cells recognizing epitopes of viral proteins that are conserved between the priming and the challenge virus do preexist. In this case, viral infection is not prevented, resulting in infected cells presenting viral peptides on MHC-II. The final column indicates the major interactions through which antiviral CD4 T cells can contribute to heterosubtypic immunity after recognizing the viral antigens presented by MHC-II.

Influenza A virus (IAV) represents a global health burden despite a highly coordinated worldwide vaccine program. The biggest current challenge in generating vaccine-induced protection is the annual reformulation of the vaccine that is required to target the predominant IAV strains that are predicted to circulate. The primary goal of vaccination is to promote protective neutralizing antibodies that recognize the major surface glycoproteins, hemagglutinin (HA) and neuraminidase (NA). This mode of protection is known as ‘homotypic’ immunity and stops the attachment of viral particles to target cells, thereby preventing infection and disease (Figure 1). A major weakness of this approach is that vaccine-primed antibodies can often be an imperfect match for circulating IAVs, due to incorrect annual predictions, for example. Indeed, HA and NA represent ‘moving targets’ for the antibody response due to antigenic shift and antigen drift [1]. There is thus much effort focused on developing improved ‘universal’ vaccines that can protect across diverse annual and even pandemic IAV strains [2,3,4]. Rather than focusing on immune responses against the variable portions of HA and NA to target specific IAV strains, a universal vaccine must instead target highly conserved epitopes that are shared between IAV strains. One approach towards this goal is focusing on the generation of ‘broadly neutralizing’ antibodies effective across different IAV strains by targeting more conserved parts of the HA and NA, like the stalk region, versus the more variable globular head region.

An alternative and not necessarily mutually exclusive approach to providing a broadly protective IAV vaccine is to prime antiviral T cells. In addition to recognizing strain-specific viral epitopes, T cells recognize viral peptides that are highly conserved between disparate IAV strains. These epitopes are commonly derived from ‘internal’ viral proteins, which are under far less immune pressure by antibodies to mutate compared to HA and NA [5]. Such T cell responses have been shown to mediate multiple mechanisms of protection underlying what is termed ‘heterosubtypic’ immunity [6]. Heterosubtypic immunity was first demonstrated in mice primed with one strain of IAV, then challenged several weeks or months later with a lethal dose of an IAV expressing a different HA/NA combination. Unlike neutralizing antibodies that can prevent infection, T cells can only recognize peptides derived from viral proteins that are presented by MHC molecules on infected cells, including lung epithelial cells [7], as well as on antigen-presenting cells and B cells (Figure 1). T cell responses thus cannot prevent initial infection [5], but instead, heterosubtypic immunity operates largely through T cell killing of infected cells, the release of diverse soluble factors from activated T cells, and the recruitment and regulation of other innate and adaptive cell types during the antiviral response. In the case of CD4 T cell-driven protection, these interactions require MHC-II-dependent interactions either with infected cells [8] or with antigen-presenting cells or B cells that have taken up viral proteins through various means (Figure 1).

Experimental [9,10] and clinical [11,12,13] studies support that CD4 T cells are a key component of optimal heterosubtypic immunity against IAV. A hallmark of CD4 T cells in general is that, once activated, a cell can differentiate into one of several different effector cell subsets directed by inflammatory factors in the priming environment. These subsets are labeled with a T-helper (Th) designation and are marked by distinct functional attributes from one another, as we will discuss below [14]. Most antiviral CD4 T cells display hallmarks of Th1 cells [15], but work using ever more sophisticated mouse models of IAV infection over the last two decades or so have revealed important contributions from other well-defined Th subsets, as well as from some CD4 T cell effectors that are not as easily binned. Here, we review these findings and discuss how they have improved the field’s understanding of the ways in which CD4 T cells can impact outcomes of IAV infection. Equally important, we will discuss how the murine IAV model continues to provide valuable insight into mechanisms controlling the effector and memory fates of CD4 T cells. An overarching theme uniting these studies is that an important goal for next-generation vaccines will be to prime a CD4 T cell landscape comprised of many distinct subsets that, in combination, synergize to provide maximal protection. This goal is encapsulated well by the Latin phrase *ex pluribus unum*: out of many, one.

## 2. Delineating Core Elements of CD4 T Cell Responses against IAV

Analysis of how CD4 T cells respond to IAV infection began in the late 1980s with assessments of the antigen specificity of MHC-II-restricted IAV-specific T cell clones in mice and humans. Studies with mice revealed broad reactivities of different clones derived from the spleens of IAV-primed mice against several different viral proteins [16]. Transferring CD4 T cell clones recognizing both conserved internal viral proteins and variable proteins like HA into syngeneic T cell-deficient hosts demonstrated help for virus-specific antibody production upon IAV infection [17], consistent with CD4 T cell-associated B-cell helper functions. Experiments over the next few years asking whether the adoptive transfer of different IAV-specific CD4 T cell clones could protect naïve mice against an otherwise lethal dose of IAV, for which they were specific, yielded mixed results depending on the clone [18,19,20,21]. This uneven set of early results may have been due to the different Th subset polarization statuses of different clones, as seminal work identifying functionally distinct Th1 vs. Th2 CD4 T cell clones was not published until around this same period [22]. Indeed, studies published in 1994 showed that IAV-specific Th1-polarized clones could protect naïve mice against lethal IAV infection while Th2 clones could not and instead promoted more severe disease [23]. The lack of protection against IAV from Th2-polarized CD4 T cells has been a consistent finding in the field.

Around the same time, work from Doherty’s group found that the depletion of CD4^+^ cells in mice prior to IAV infection delayed viral clearance by up to 4 days, but that the mice still survived [24,25]. Using mouse models to eliminate antiviral CD8 T cell responses also revealed relatively intact viral clearance, which was, however, abrogated if CD4 T cells were also depleted [26]. Further work revealed that CD4 T cells alone could not efficiently clear IAV, as B cell-deficient mice depleted of CD8 T cells (thus leaving only CD4 T cells as the major adaptive immune cell subset) were far more susceptible to infection-induced death than B cell-deficient mice treated with an isotype control antibody [27,28]. It should be noted that B cell-deficient mice depleted of CD4^+^ cells were marked by much higher mortality than B cell-deficient mice without CD4 T cell depletion [29], supporting the conclusion that responses from CD4 and CD8 T cells with B cells synergize to promote optimal IAV clearance. Together, these earlier studies thus revealed that while properly polarized CD4 T cells could be highly protective, especially in synergy with antiviral CD8 T cell or B cell responses, CD4 T cells are not an absolute requirement for IAV clearance in the presence of an otherwise intact adaptive immune response.

**Figure 2:**
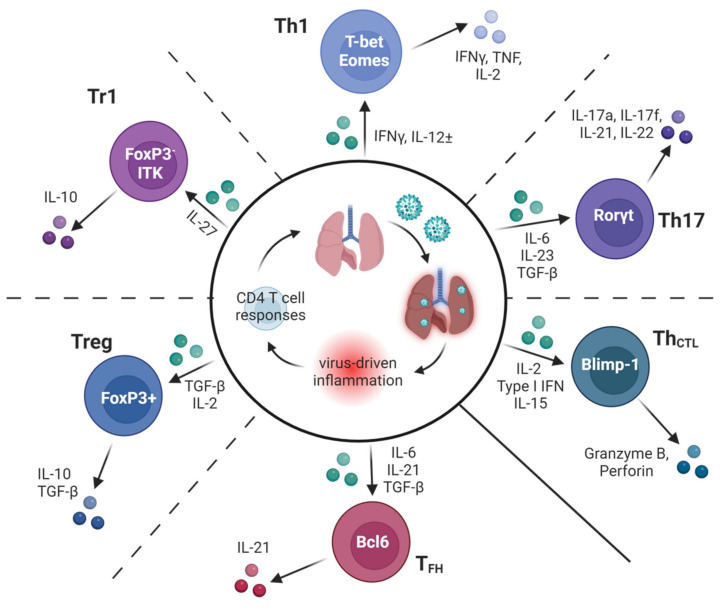
Many subsets of specialized CD4 T cells contribute to optimal protection against IAV. A schematic of the diverse types of CD4 T cell responses found to impact the outcomes of IAV infection in murine models. Key inflammatory factors induced by IAV infection known to guide the differentiation of subsets are shown in green, along with hallmark transcriptional regulators and secreted products of each subset. Dotted lines between subsets represent some of the possible plasticity reported in the literature between adjacent subsets.

Subsequent work in the 1990s sought to characterize mechanisms by which CD4 T cells protect against IAV. Surprisingly, given that ‘Th1′ inflammation predominates during IAV infection and that early protection studies identified Th1 clones as protective, IFNγ was not found to be needed for successful IAV clearance in studies using IFNγ-deficient mice [30] or the neutralization of IFNγ by in vivo antibody treatment [31]. However, the neutralization of IFNγ by in β2-microglobulin-deficient mice (which lack CD8 T cells) did delay viral clearance [31], suggesting that IFNγ production by CD4 T cells may become more important in the absence of other major antiviral lymphocyte subsets. This conclusion is supported by studies in which the transfer of IFNγ-deficient Th1-polarized memory CD4 T cells was shown to protect wildtype mice, as well as B cell-deficient or CD8 T cell-deficient mice, from an otherwise lethal IAV challenge, but the cells could not promote viral clearance in host mice lacking T cells and B cells [32]. These outcomes are perhaps surprising as IFNγ is an absolute requirement for protection in several other models of viral, bacterial, and parasitic infections dominated by a Th1 response signature [33]. In the following sections, we will highlight findings showing that several distinct subsets of CD4 T cell effectors, including Th1, Th17, follicular helpers (T_FH_), regulatory T cell subsets, and cytotoxic CD4 T cells (Th_CTL_) (Figure 2) can all contribute in distinct ways to immunity against IAV.

## 3. Th1 Responses against IAV

Full Th1 differentiation in vitro requires IL-12 and IFNγ-mediated signals that operate through STAT4 and STAT1, respectively, to maximize the expression of the Th1 ‘master regulator’ transcription factor, T-bet. This transcriptional program supports not only strong Th1 induction but also restricts polarization towards other specialized Th subsets. Th1 criteria, highlighted by strong IFNγ production and T-bet expression, characterize the majority of IAV-primed CD4 T cells present both in lymphoid organs and in the lung. However, early experiments using adoptive transfer of naïve TcR transgenic CD4 T cells recognizing an IAV antigen to unprimed mice prior to infection revealed remarkable heterogeneity between effector cells derived from the donor cells in the spleen, mediastinal lymph node, and lung, and even within the cohort of effector cells present at each tissue site [34]. This heterogeneity between tissues was even more evident in experiments comparing the gene expression of sort-purified IAV-specific TcR transgenic populations at the peak of the antiviral response [35]. Nevertheless, and in agreement with earlier studies using long-term CD4 T cell clones, the transfer of relatively homogenous effector CD4 T cells generated in vitro from naïve IAV-specific transgenic cells under Th1-polarizing conditions (including blocking antibodies against IL-4 and recombinant IL-12 to drive the initial stages of Th1-differentiation) provided strong protection to unprimed mice against an otherwise lethal challenge dose [36] but did not require IFNγ production from the transferred effector cells [7]. Perhaps surprisingly, the identification of discrete protective mechanisms required for Th1 effector cells to protect against IAV remains largely elusive.

## 4. Does IAV Prime True ‘Th1′ Cells?

We recently used the in vitro-generated CD4 T cell effector transfer model to ‘strip away’ aspects of the Th1-ness of effector cells by using cells knocked out for major transcription factors driving Th1 fate, including T-bet, Eomesodermin (Eomes), STAT1, and STAT4. We initially compared the protective capacity of T-bet-deficient vs. WT OT-II cells primed in Th1 conditions after their transfer to unprimed mice that were infected with an IAV expressing the epitope recognized by the OT-II TcR. The protection and viral control afforded by both types of Th1 effectors were equivalent despite a reduction in the production of Th1-associated cytokines by the T-bet-deficient cells [37]. While at first glance this might suggest that Th1-mediated protection against IAV is independent of T-bet, many of the T-bet-deficient effectors developed strong Th17 attributes, and, as will be discussed below, Th17 responses have also been increasingly associated with positive outcomes of IAV infection. Teasing out the contributions of residual ‘Th1-ness’ from the emergent ‘Th17-ness’ or other protective attributes in these situations is difficult. STAT1-deficient OT-II cells primed in Th1 conditions also displayed reduced Th1 effector functions and reduced T-bet expression but retained similar protective capacity as WT effectors [38]. However, our results showed that STAT1 expression by antiviral CD4 T cells was required to prevent their deletion during IAV infection by NK cells [38]. These results highlight an absolute need for STAT1 signaling to promote productive antiviral Th1 responses during IAV infection.

Pioneering mouse studies found a role for IL-12 in the early coordination of innate immunity upon infection, but IL-12 signals did not impact the ability of IAV-primed CD4 T cells to produce IFNγ [39]. These findings were later extended with an analysis of STAT4-deficient mice that revealed only a marginal loss of Th1 identity by CD4 T cells responding to IAV infection [40]. As IAV-induced inflammation is altered in STAT4-deficient vs. wildtype mice, which could indirectly affect T cell priming, we compared responses to IAV by wildtype and STAT4-deficient CD4 T cells responding in otherwise wildtype mice. We also found minimal impacts of STAT4 expression on the magnitude or general Th1 character of the response [38]. However, the treatment of mice with recombinant IL-12 during IAV infection dramatically enhanced the Th1 imprint of responding wildtype but not STAT4-deficient cells [38]. These observations may help to explain the potent adjuvant effect of IL-12 found in studies assessing vaccine-induced immunity against IAV, including T cell-dependent heterosubtypic protection, by strengthening the Th1 arm of the antiviral response [41,42,43]. Indeed, a few observations have found that IFNγ production is required for optimal protection of mice against IAV [44,45,46], and it is tempting to speculate that these outcomes may be at least partially due to CD4 T cells with stronger Th1 imprints being generated. We speculate that strong IL-12 signaling may thus amplify the relative importance of Th1 attributes, like IFNγ, in the protective impact of antiviral CD4 T cells while perhaps reducing the importance of contributions from other effector subsets.

The results discussed above indicate that IAV infection does not induce a maximal Th1 imprint in responding CD4 T cells, as is seen in responses against other intracellular pathogens, particularly those driving phagosomal infections in macrophages [47]. It is possible that a weaker Th1 imprint is important to restrain the otherwise too-damaging immunopathological consequences of antiviral CD4 T cells in the lung. In support of this hypothesis, accumulating data indicate the capacity of IFNγ to exacerbate disease symptoms via several different mechanisms [48,49,50,51]. It is also possible that a more limited Th1 induction is important for the striking heterogeneity in effector phenotypes (Figure 2) that characterize the effector and memory CD4 T cell pools [52], which will be discussed below and which may be required for optimal protection. Given the correlation of CD4 T cells expressing higher levels of T-bet [37,53] and producing more IFNγ [54] and less IL-2 [55] with reduced memory capacity, it is also possible that a submaximal Th1 program is important to establish optimal CD4 T cell memory following the resolution of IAV infection. Indeed, T-bet expression by CD4 T cells appears to restrict the generation of circulating and lung tissue-resident cells (T_RM_) primed by IAV infection [37,56].

## 5. Th17 Responses

Virus-specific T cells producing IL-17 during IAV infection [57] and the protective capacity of in vitro-polarized Th17 cells [58] in mice were first reported in 2009. Protection mediated by IAV vaccine-primed Th17 cells has also been demonstrated in mice [59]. A separate set of observations directly correlated IL-17 production during IAV infection with increased lung immunopathology in mice [60], a relationship that has been observed in many subsequent studies [59,61,62,63,64]. On the other hand, IL-17 has been shown to be an important component promoting optimal B cell responses in the lung during IAV infection in mice [65,66]. More recent studies have identified IAV-specific Th17 lung tissue-resident memory cells and demonstrated their protective capacity during recall infection [67,68]. It thus appears that, just as for the production of IFNγ by Th1 cells discussed above, the production of IL-17 by Th17 cells responding to IAV must be well-calibrated to avoid excess immunopathology.

Two challenges, however, in stating with confidence that Th17 cells can mediate protective responses against IAV are introduced by their relatively small representation in the IAV effector CD4 T cell pool in wildtype mice [69] and their well-documented functional plasticity leading to the adoption of Th1 attributes in Th1-associated inflammatory settings [70]. This plasticity can give rise to a mixed Th1/Th17 response on the population level, as seen in the response of T-bet-deficient cells to IAV, discussed earlier. In our studies on the regulators of Th1-polarization during IAV infection, we found that virus-specific CD4 T cells lacking T-bet and Eomes gave rise to effectors with strong Th17 imprints *without* concomitant Th1 functionality [71]. When these T-bet/Eomes double knockout (DKO) cells were used to generate Th17 effectors in vitro and then transferred to unprimed wildtype mice to test their protective capacity during IAV infection, the effectors not only held their Th17 phenotype but the imprint was boosted, resulting in nearly 80% of the cells expressing the Th17 ‘master regulator’ Rorγt. This model thus allows for more definitive conclusions regarding the protective capacity of Th17 responses in the setting of IAV. Indeed, Th17 effectors generated from DKO precursors in vitro, which were unadulterated by Th1 attributes when assessed in vivo, were equally protective to Th1-polarized wildtype effector cells when transferred to naïve mice challenged with a lethal dose of IAV. In agreement with known drivers of Th17 differentiation in vitro, the blockade of IL-6 and TGFβ in mice during IAV infection abrogated the development of effectors derived from DKO CD4 T cells expressing Rorγt and Th17 cytokines [71]. Interestingly, the treatment of mice receiving transferred non-plastic Th17-polarized cells with IL-17 neutralizing antibodies did not impact their ability to protect [37]. Less is known about how other cytokines produced by Th17 cells, including IL-17F, IL-21, and IL-22, affect outcomes of infection, though a generally positive role for the latter two factors during IAV infection has been reported [72,73].

The endogenous antiviral T cell response in DKO mice not expressing a TcR transgene is also Th17 (and Tc17)-polarized, with virtually no Th1/Tc1 signature. Furthermore, we showed that protection against an H3N2 heterosubtypic challenge in DKO mice primed by an H1N1 virus could be mediated by either memory CD4 or CD8 T cells [71], consistent with previous observations of robust heterosubtypic immunity in wildtype mice being mediated by either CD4 or CD8 memory cells [74], and also consistent with a protective impact of Tc17 CD8 T cells against IAV [57]. Interestingly, the same DKO mice rapidly die following LCMV infection due to T cell-dependent Th17-driven inflammation [75]. More research is thus required to understand the mechanisms by which Th17 responses impact positive versus negative outcomes during infection with IAV and viral infection more generally [76,77,78].

Beyond Th17-mediated antiviral impacts, targeting a Th17 component in the IAV-specific CD4 T cell landscape may have other benefits. For example, vaccine priming of Th17 cells might lead to improved memory longevity, as evidence from many studies indicates that Th17-polarized effector cells are better able to survive acute contraction than Th1 effectors [79]. This consideration may be particularly relevant for IAV-specific memory T_FH_ cells [80], which, as will be discussed, are important for optimal long-term humoral immunity.

Another way in which incorporating Th17 responses into IAV vaccine strategies may promote positive outcomes is by ensuring inflammatory flexibility in the lung to avoid bacterial superinfection, the most serious comorbidity of IAV infection [81,82]. Enhanced neutrophil responses are commonly associated with Th17 activation, and neutrophils have been found to mediate protective impacts in some models of IAV infection [83]. Importantly, Th17-associated cytokines and neutrophils are strongly linked to resistance to bacterial superinfection in mice following IAV infection caused by pathogens like *Streptococcus pneumoniae* [81,84]. Indeed, studies using T-bet-deficient mice found that the mixed Th1/Th17 response following IAV infection correlates with IL-17- and neutrophil-dependent protection from otherwise lethal superinfection with *Streptococcus pneumoniae* [85]. Further studies are required to determine the extent to which antiviral Th17 cells responding in a wildtype environment can promote superinfection resistance.

## 6. Cytotoxic CD4 T Cells (Th_CTL_)

That some CD4 T cells develop cytotoxic function during IAV infection, and that this correlates with protection, was noted in early work analyzing virus-specific CD4 T cell clones [23]. Mouse studies have since found decreased protection afforded by CD4 T cell effectors deficient for perforin [32,45], and preexisting cytotoxic CD4 T cells have been correlated with protection against IAV infection in humans [11]. The benefits of priming an MHC-II restricted Th_CTL_ subset in addition to CD8^+^ T cells may include broadening the antigenic determinants able to promote the killing of ‘factories’ producing new virions and/or by serving as a backup in cases where CD8^+^ T cell killing may be compromised. For example, IAV can downregulate the expression of MHC-I on infected epithelial cells and other cell types [86]. Supporting the concept that Th_CTL_ may impact immune responses against IAV largely through the killing of infected cells, Th_CTL_ cells are mostly found in the lung (the site of infection), and the differentiation of this subset appears to occur locally in the lung well after the initial priming in secondary lymphoid organs [87].

The relationship between the programming of cytotoxic potential in CD4 T cells and Th1 imprinting is not entirely clear. Initial studies into this question found that the key Th1-inducing cytokines IFNγ and IL-12 were not required to prime Th_CTL_ in vitro but that IL-2 was a critical factor promoting perforin-dependent cytotoxic function [88]. Earlier work with IAV-primed clones also found IL-2 to promote FasL/Fas-dependent cytotoxicity [89]. The importance of IL-2 in promoting Th_CTL_ is further supported by more recent observations indicating that the transcription factor Alios is a key factor in regulating IL-2 sensitivity, with cells deficient for Alios gaining more hallmarks of cytotoxic function, including the expression of the transcription factor Eomes during IAV infection [90]. Interestingly, while Eomes expression is broadly associated with CD4 T cell killing in diverse settings [91], we found the in vivo cytotoxic activity of T-bet/Eomes double knockout effector cells primed by IAV to be largely intact when compared to wildtype CD4 T cells with the same TcR specificity [71]. These discordant results may reflect that multiple, distinct transcriptional programs are able to support the acquisition of cytotoxic function by CD4 T cell effectors.

Another important signal for Th_CTL_ differentiation appears to be type I IFN. Sun and colleagues found that STAT2-dependent type I IFN signals synergized with STAT5-dependent IL-2 signals to increase the expression of the cytolytic factor granzyme B by CD4 T cells during IAV infection [92]. These studies also found a requirement for the transcription factor Blimp-1 in promoting Th_CTL_, a finding confirmed by studies by Swain’s group, who also identified NKGA/C/E as a reliable phenotypic marker of IAV-primed Th_CTL_ [93]. More recent work identifies IL-15, driven by type I IFN during IAV infection, as another important signal to optimize Th_CTL_ development in the lung [87]. Like IL-2, IL-15 signals through CD122 and STAT5. As CD4 T cells responding to IAV in the lung make far less IL-2 than effectors in secondary lymphoid organs [35], it is tempting to speculate that local IL-15 serves as a replacement for IL-2 in directing Th_CTL_ development in the lung.

## 7. Follicular Helper Cells (T_FH_)

Given the critical importance of long-lived neutralizing antibodies for homotypic immunity against IAV, much investigation has focused on the molecular interactions required for CD4 T cell help for B cells during IAV infection. Specialized programming associated with T_FH_ is required for optimal CD4 T cell-dependent Ab production as shown with experiments comparing the responses of wildtype and SLAM-associated protein (SAP)-deficient CD4 T cells. While SAP-deficient cells formed effector populations characterized by Th1 functional criteria that were similar to wildtype cells, their ability to help B cells was highly compromised, as shown by the slower kinetics and reduced amounts of antiviral IgG production [94]. This is consistent with a requirement for SAP expression by CD4 T cells for optimal humoral responses against T cell-dependent antigens more generally [95]. Further studies have shown that signals found to be required to generate T_FH_ in other settings, namely IL-6 and IL-21, are required for T_FH_ development and optimal antibody responses following IAV infection [96]. In contrast, IL-2 signals seem to restrict T_FH_ differentiation [97]. Evidence suggests that TGFβ, which is activated by the IAV neuraminidase [98], is important in suppressing the expression of the high affinity IL-2 receptor by CD4 T cells activated by IAV, thus helping to promote T_FH_ differentiation [99]. IL-2 signaling thus seems to be an important regulatory switch that promotes Th_CTL_ fate, as discussed above, while restricting T_FH_ fate.

A further body of evidence indicates that, as compared to the acquisition of Th1 or Th17 attributes, which occurs relatively early during IAV infection, T_FH_ differentiation occurs during a late-acting window. T_FH_ seems to develop in the draining lymph node after interacting with migratory dendritic cells that traffic from the lung only at around 6 days after infection [100]. This timeframe is in agreement with more recent work that found that no particular subset of antigen-presenting cells was required to promote T_FH_ differentiation [101], as opposed to studies in other models that have demonstrated an obligate role for migratory CD11b^+^ dendritic cells in promoting T_FH_ differentiation [102]. Recent findings also indicate that the expression of the transcription factor Tox2 is important for IAV-primed T_FH_ to maintain their programming, even into memory [103]. Perhaps surprisingly, given the correlation of Th2 cytokines with worsened outcomes of IAV infection, IL-4 production by IAV-primed T_FH_ has been shown to be required for optimal germinal center responses [104].

Interestingly, mice deficient in the expression of Bcl6, the hallmark ‘master regulator’ of T_FH_ differentiation, have been shown to generate largely intact IgG responses after IAV vaccination, which can protect against an otherwise lethal homotypic challenge. This antibody response was nevertheless shown to require CD4 T cell-dependent, CXCR3-expressing Th1 cells and their production of IFNγ and IL-21 [105]. While this suggests that multiple modes of CD4 T cell help can promote a protective level of humoral immunity, the detection of IAV-specific T_FH_ strongly correlates with improved antiviral Ab responses in clinical studies [106,107,108], highlighting the value of T_FH_ indication as a target of vaccination.

## 8. CD4 T Cell Help for Antiviral CD8 T Cell Responses

Several studies have shown that CD4 T cells have a strong impact on aspects of the CD8 T cell response against IAV. While optimal help for B cell responses seems to require a specialized subset of CD4 T cell effectors (T_FH_), it is not clear that this is the case for CD4 T cell help for CD8 T cells. However, recent studies have shown an important role for IL-21-dependent help for CD8 T cell responses against IAV in the lung by a population of T_FH_-like resident memory cells [109]. Most data indicate that CD8 T cell responses generated against IAV in the absence of CD4 T cell help are similar during the effector phase but that a reduction in CD8 T cell memory formation is seen. The un-helped CD8 T cells are characterized by several changes versus helped CD8 T cells including alterations in metabolic pathways [110] and transcription factor expression including higher levels of T-bet that correlate with a more terminal fate [111]. Analysis of the mode of CD4 help involved supports that their activation of APC through the delivery of CD40 ligand signals licenses APC to deliver critical pro-memory signals to the CD8 T cells [112]. This general mechanism may also act to promote optimal CD4 T cell responses during IAV infection [113]. However, stimulation through NKG2D on CD8 T cells has been shown to be able to substitute for CD4 T cell help in IAV-infected mice [114], suggesting redundancy in pathways able to promote effective primary and secondary CD8 T cell responses against IAV.

## 9. Regulatory CD4 T Cells

FoxP3^+^ regulatory CD4 T cells (Treg) are detected during IAV infection both in the lungs and in secondary lymphoid organs [115,116]. Tregs have been shown to restrict effector T cell responses in the lung during primary IAV infection in some studies [116] and to temper innate inflammatory responses [117,118]. Treg depletion during heterosubtypic infection has been shown to have broader impacts, including increasing lung pathology and decreasing pulmonary function [119]. The more severe outcomes of Treg depletion during heterosubtypic challenge may be due to the more potent suppressive capacity of IAV-primed memory versus primary Tregs more generally [120] coupled with a need for increased regulation to keep potentially more damaging larger secondary effector T cell responses in check. Beyond traditional regulatory function, recent studies have identified novel ways in which Tregs can impact the outcome of IAV infection. These include the optimization of epithelial repair [121] and helping to optimize T_FH_ development by limiting local IL-2 signals, which, as discussed earlier, can interfere with T_FH_ differentiation [122]. Furthermore, FoxP3^+^ CD4 T cells co-expressing the T_FH_ hallmarks Bcl6 and CXCR5 have been shown to be critical in optimizing IAV-primed antibody responses in germinal centers [123], though the mechanisms involved are not yet clear.

CD4^+^ FoxP3^−^ IL-10-producing regulatory T cells have also been well-described during IAV infection. In fact, we found that most IL-10 produced in the lungs of IAV-infected mice is derived from FoxP3^-^ CD4 T cell effectors [58], often termed ‘Tr1′ cells, consistent with a more recent study that described an unexpected degree of heterogeneity within the IAV-primed Tr1 cohort [124]. Interestingly, IL-10′s impacts during IAV infection appear to be variable: both improved outcomes [58,125] in the absence of IL-10 and more severe disease [124,126] have been reported. In our studies, the improved outcomes of IAV infection in mice deficient for IL-10 or treated to block IL-10 signaling correlated with the emergence of a much stronger antiviral Th17 response [58], consistent with other reports of IL-10 negatively regulating Th17-associated cytokine expression [127]. It is likely that the positive versus negative impacts of IL-10 on the outcome of IAV infection can be impacted by subtle changes in the constituents of heightened inflammation between experimental models. Beyond a regulatory role during IAV infection, IL-10 production by CD4 T cells may, somewhat paradoxically, be required to support the development of prototypical Th1-like antiviral function by countering the suppressive impact of TGFβ produced early during IAV infection [128]. We found that STAT1 expression by IAV-primed CD4 T cells is critical for their ability to develop the capacity to produce IL-10 [38]. This is consistent with the requirement for the STAT1-dependent cytokine IL-27 to generate IAV-primed Tr1 cells [124]. Expression of the TEC kinase ITK also seems to be important for optimal development of IAV-primed Tr1 cells [129].

## 10. Outlook for Vaccine-Mediated CD4 T Cell Protection against IAV

The studies discussed above highlight the tremendous potential of CD4 T cells to impact outcomes of IAV infection through multiple independent mechanisms that are mediated by distinct subsets of effector cells. However, several major challenges remain in translating this potential into improved vaccines. First, to harness the multiple modes of action discussed here, a vaccine must be able to reproduce the surprisingly broad heterogeneity within the CD4 T cell effector landscape that is seen during IAV infection. It is likely that ever-increasing heterogeneity within IAV-primed CD4 T cells will be uncovered by more powerful analysis platforms, as shown recently with the advanced interrogation of human CD4 T cells [130]. One approach to achieve this is to formulate vaccines with multiple adjuvants that are each able to promote the polarization of specialized effector cells [131]. But, perhaps the most straightforward route to replicate IAV-induced CD4 T cell effector landscapes is to use attenuated IAV vaccine platforms that encode all of the innate triggers of virulent IAV and that target local responses in the airways [132].

Secondly, an ideal vaccine should aim to replicate the patterns of the location of memory T cells primed by natural infection. As it is increasingly clear that lung T_RM_ cells are critical in T cell-mediated protection against respiratory viruses [133], and CD4 T_RM_ cells in particular have been shown to be highly protective in animal models [134,135], the seeding of this subset is highly desirable. Due to their location at the site of infection, CD4 T_RM_ can be activated and drive protective inflammatory responses in the lung prior to activation of memory T cells in secondary lymphoid organs [136,137]. As supported by animal studies, attenuated IAV vaccines delivered intranasally represent a straightforward approach to target the priming of protective airway-resident memory T cells [134]. However, it is important to note that the performance of licensed live attenuated IAV vaccines based on cold-adapted IAV backbones in humans has been uneven. This is highlighted by the very low efficacy found during the 2013–2015 flu seasons among children, a group in which live attenuated vaccines have generally been found to have high efficacy [138]. Several potential issues have been raised in explaining the relatively low efficacy of live attenuated IAV strains including issues with the parental vaccine strain resulting in decreased infectivity, a high concentration of non-infective viral particles, and issues related to thermal degradation of the vaccine in the field. The optimization of live attenuated vaccines is thus an active field of research [139].

Third, the signals that promote optimal CD4 T cell memory formation are incompletely understood. We have shown that antigen recognition by CD4 T cells responding to IAV during the effector phase is important to induce autocrine IL-2 signaling, which helps promote the improved memory fitness of the effector cells [55,140]. Importantly, this phase of the response occurs several days after initial CD4 T cell priming. Providing more antigen to stimulate antiviral CD4 T cells at this later phase of the response, or more IL-2 signals, to boost memory does not seem to restrict the development of specialized subsets like T_FH_ or TH_CTL_ [141]. This suggests that vaccine platforms able to improve antigen persistence in order to boost CD4 T cell memory may still allow for the priming of a heterogeneous effector CD4 T cell landscape. Importantly, we found that increasing antigen stimulation of CD4 T cells during this later effector phase of the response primed by a cold-adapted IAV vaccine strain boosted memory formation, especially in the lung [140]. But increasing the magnitude or duration of vaccine-induced T cell stimulation may also carry risks. For example, IL-2 production by CD4 T cells recognizing viral antigens can contribute to IAV-induced immunopathology [142,143]. Earlier signals that operate during CD4 T cell priming have also been shown to be important in directing memory fates [144], but their impacts during IAV infection are not clearly understood. Delineating the full set of signals regulating the efficiency by which antiviral CD4 T cells can realize memory potential is thus a critical goal to facilitate vaccine approaches to optimize T cell immunity.

Several other considerations are also important. For example, do specific viral proteins or epitopes of proteins mark ideal vaccine targets? Work by Sant and colleagues indicates that HA-specific CD4 T cells mark T_FH_ as able to best promote HA-specific antibody responses versus T_FH_ recognizing other viral proteins [145]. Their studies have also made clear the challenge of overcoming preexisting virus-specific T cells to populate new TcR specificities in the memory CD4 T cell pool [146]. Indeed, complex patterns of preexisting cellular and humoral immunity against IAV between individuals induced by annual seasonal infections and/or vaccinations are likely to impact live attenuated vaccine efficacy. This is primarily due to the need for the vaccine to evade neutralizing antibodies, and perhaps heterosubtypic T cell responses, to induce a level of infection that is sufficient to stimulate robust new immune responses [147]. In support of the negative impact of preexisting immunity on the induction of new memory T cells against IAV, we have shown that preexisting neutralizing antibodies somewhat restrict the priming of antiviral CD4 T cells after IAV infection in mice but severely restrict their memory potential [148]. Similar findings have been made assessing CD8 T cell responses induced by live attenuated vaccination in mice in the face of preexisting immunity [149]. Novel approaches to overcome preexisting immunity may be critical for vaccination to prime durable T cell immunity against IAV.

Finally, the definition of more incisive correlates of protective CD4 T cells is needed. IFNγ production by CD4 T cells responding to IAV is almost universally used as a functional readout of clinical and vaccine studies [150] but is not found to be required universally for their protective impact in animal models or humans. Furthermore, the production of IFNγ is not necessarily a hallmark of all of the specialized subsets found to affect the outcomes of infection, as discussed here. The detection of multicytokine-producing CD4 T cells may be more informative, as effectors able to produce IFNγ together with IL-2 and TNF are correlated with improved protection in some mouse studies [35,151]. However, a full evaluation of the protective capacity of any vaccine-induced CD4 T cell response is likely to require an even broader analysis able to interrogate the diverse functions of the several subsets described here that together comprise the anti-IAV CD4 T cell response.

## 11. Conclusions

Research over the past few decades has solidified the protective impact of CD4 T cells during IAV infection but it has also revealed unexpected complexities in the antiviral CD4 T cell landscape. Key shorter-term goals for the field include the identification of critical functional components underlying optimal CD4 T cell responses against IAV and signals controlling the development of lung-resident and systemic memory CD4 T cell pools. Advances on these fronts are prerequisite for the development of universal IAV vaccines that leverage diverse populations of CD4 T cells as a key element of long-lived heterosubtypic protection.

## References

[1] Kim H., Webster R.G., Webby R.J. (2018). Influenza Virus: Dealing with a Drifting and Shifting Pathogen. Viral Immunol..

[2] Corder B.N., Bullard B.L., Poland G.A., Weaver E.A. (2020). A Decade in Review: A Systematic Review of Universal Influenza Vaccines in Clinical Trials during the 2010 Decade. Viruses.

[3] Nachbagauer R., Palese P. (2020). Is a Universal Influenza Virus Vaccine Possible?. Annu. Rev. Med..

[4] Paules C.I., Sullivan S.G., Subbarao K., Fauci A.S. (2018). Chasing Seasonal Influenza—The Need for a Universal Influenza Vaccine. N. Engl. J. Med..

[5] Powell T.J., Strutt T., Reome J., Hollenbaugh J.A., Roberts A.D., Woodland D.L., Swain S.L., Dutton R.W. (2007). Priming with cold-adapted influenza A does not prevent infection but elicits long-lived protection against supralethal challenge with heterosubtypic virus. J. Immunol..

[6] La Gruta N.L., Turner S.J. (2014). T cell mediated immunity to influenza: Mechanisms of viral control. Trends Immunol..

[7] Brown D.M., Dilzer A.M., Meents D.L., Swain S.L. (2006). CD4 T cell-mediated protection from lethal influenza: Perforin and antibody-mediated mechanisms give a one-two punch. J. Immunol..

[8] Schulman J.L., Kilbourne E.D. (1965). Induction of Partial Specific Heterotypic Immunity in Mice by a Single Infection with Influenza a Virus. J. Bacteriol..

[9] Sridhar S. (2016). Heterosubtypic T-Cell Immunity to Influenza in Humans: Challenges for Universal T-Cell Influenza Vaccines. Front. Immunol..

[10] Altenburg A.F., Rimmelzwaan G.F., de Vries R.D. (2015). Virus-specific T cells as correlate of (cross-)protective immunity against influenza. Vaccine.

[11] Wilkinson T.M., Li C.K., Chui C.S., Huang A.K., Perkins M., Liebner J.C., Lambkin-Williams R., Gilbert A., Oxford J., Nicholas B. (2012). Preexisting influenza-specific CD4^+^ T cells correlate with disease protection against influenza challenge in humans. Nat. Med..

[12] Mettelman R.C., Souquette A., Van de Velde L.A., Vegesana K., Allen E.K., Kackos C.M., Trifkovic S., DeBeauchamp J., Wilson T.L., St James D.G. (2023). Baseline innate and T cell populations are correlates of protection against symptomatic influenza virus infection independent of serology. Nat. Immunol..

[13] Tsang T.K., Lam K.T., Liu Y., Fang V.J., Mu X., Leung N.H.L., Peiris J.S.M., Leung G.M., Cowling B.J., Tu W. (2022). Investigation of CD4 and CD8 T cell-mediated protection against influenza A virus in a cohort study. BMC Med..

[14] Zielinski C.E. (2023). T helper cell subsets: Diversification of the field. Eur. J. Immunol..

[15] Swain S.L., McKinstry K.K., Strutt T.M. (2012). Expanding roles for CD4^+^ T cells in immunity to viruses. Nat. Rev. Immunol..

[16] Mills K.H., Skehel J.J., Thomas D.B. (1986). Extensive diversity in the recognition of influenza virus hemagglutinin by murine T helper clones. J. Exp. Med..

[17] Scherle P.A., Gerhard W. (1986). Functional analysis of influenza-specific helper T cell clones in vivo. T cells specific for internal viral proteins provide cognate help for B cell responses to hemagglutinin. J. Exp. Med..

[18] McDermott M.R., Lukacher A.E., Braciale V.L., Braciale T.J., Bienenstock J. (1987). Characterization and in vivo distribution of influenza-virus-specific T-lymphocytes in the murine respiratory tract. Am. Rev. Respir. Dis..

[19] Askonas B.A., Taylor P.M., Esquivel F. (1988). Cytotoxic T cells in influenza infection. Ann. N. Y. Acad. Sci..

[20] Taylor P.M., Esquivel F., Askonas B.A. (1990). Murine CD4^+^ T cell clones vary in function in vitro and in influenza infection in vivo. Int. Immunol..

[21] Scherle P.A., Palladino G., Gerhard W. (1992). Mice can recover from pulmonary influenza virus infection in the absence of class I-restricted cytotoxic T cells. J. Immunol..

[22] Mosmann T.R., Cherwinski H., Bond M.W., Giedlin M.A., Coffman R.L. (1986). Two types of murine helper T cell clone. I. Definition according to profiles of lymphokine activities and secreted proteins. J. Immunol..

[23] Graham M.B., Braciale V.L., Braciale T.J. (1994). Influenza virus-specific CD4+ T helper type 2 T lymphocytes do not promote recovery from experimental virus infection. J. Exp. Med..

[24] Eichelberger M.C., Wang M.L., Allan W., Webster R.G., Doherty P.C. (1991). Influenza virus RNA in the lung and lymphoid tissue of immunologically intact and CD4-depleted mice. J. Gen. Virol..

[25] Allan W., Tabi Z., Cleary A., Doherty P.C. (1990). Cellular events in the lymph node and lung of mice with influenza. Consequences of depleting CD4+ T cells. J. Immunol..

[26] Eichelberger M., Allan W., Zijlstra M., Jaenisch R., Doherty P.C. (1991). Clearance of influenza virus respiratory infection in mice lacking class I major histocompatibility complex-restricted CD8+ T cells. J. Exp. Med..

[27] Topham D.J., Doherty P.C. (1998). Clearance of an influenza A virus by CD4^+^ T cells is inefficient in the absence of B cells. J. Virol..

[28] Mozdzanowska K., Furchner M., Maiese K., Gerhard W. (1997). CD4+ T cells are ineffective in clearing a pulmonary infection with influenza type A virus in the absence of B cells. Virology.

[29] Mozdzanowska K., Maiese K., Gerhard W. (2000). Th cell-deficient mice control influenza virus infection more effectively than Th- and B cell-deficient mice: Evidence for a Th-independent contribution by B cells to virus clearance. J. Immunol..

[30] Graham M.B., Dalton D.K., Giltinan D., Braciale V.L., Stewart T.A., Braciale T.J. (1993). Response to influenza infection in mice with a targeted disruption in the interferon gamma gene. J. Exp. Med..

[31] Sarawar S.R., Sangster M., Coffman R.L., Doherty P.C. (1994). Administration of anti-IFN-gamma antibody to beta 2-microglobulin-deficient mice delays influenza virus clearance but does not switch the response to a T helper cell 2 phenotype. J. Immunol..

[32] McKinstry K.K., Strutt T.M., Kuang Y., Brown D.M., Sell S., Dutton R.W., Swain S.L. (2012). Memory CD4^+^ T cells protect against influenza through multiple synergizing mechanisms. J. Clin. Investig..

[33] Szabo S.J., Sullivan B.M., Peng S.L., Glimcher L.H. (2003). Molecular mechanisms regulating Th1 immune responses. Annu. Rev. Immunol..

[34] Roman E., Miller E., Harmsen A., Wiley J., Von Andrian U.H., Huston G., Swain S.L. (2002). CD4 effector T cell subsets in the response to influenza: Heterogeneity, migration, and function. J. Exp. Med..

[35] Strutt T.M., McKinstry K.K., Kuang Y., Bradley L.M., Swain S.L. (2012). Memory CD4^+^ T-cell-mediated protection depends on secondary effectors that are distinct from and superior to primary effectors. Proc. Natl. Acad. Sci. USA.

[36] Jelley-Gibbs D.M., Dibble J.P., Filipson S., Haynes L., Kemp R.A., Swain S.L. (2005). Repeated stimulation of CD4 effector T cells can limit their protective function. J. Exp. Med..

[37] Dhume K., Finn C.M., Strutt T.M., Sell S., McKinstry K.K. (2019). T-bet optimizes CD4 T-cell responses against influenza through CXCR3-dependent lung trafficking but not functional programming. Mucosal Immunol..

[38] Finn C.M., Dhume K., Prokop E., Strutt T.M., McKinstry K.K. (2023). STAT1 Controls the Functionality of Influenza-Primed CD4 T Cells but Therapeutic STAT4 Engagement Maximizes Their Antiviral Impact. J. Immunol..

[39] Monteiro J.M., Harvey C., Trinchieri G. (1998). Role of interleukin-12 in primary influenza virus infection. J. Virol..

[40] Bot A., Rodrigo E., Wolfe T., Bot S., Von Herrath M.G. (2003). Infection-triggered regulatory mechanisms override the role of STAT 4 in control of the immune response to influenza virus antigens. J. Virol..

[41] Arulanandam B.P., Mittler J.N., Lee W.T., O’Toole M., Metzger D.W. (2000). Neonatal administration of IL-12 enhances the protective efficacy of antiviral vaccines. J. Immunol..

[42] Khan T., Heffron C.L., High K.P., Roberts P.C. (2014). Tailored vaccines targeting the elderly using whole inactivated influenza vaccines bearing cytokine immunomodulators. J. Interferon Cytokine Res..

[43] Maegawa K., Sugita S., Arasaki Y., Nerome R., Nerome K. (2020). Interleukin 12-containing influenza virus-like-particle vaccine elevate its protective activity against heterotypic influenza virus infection. Heliyon.

[44] Teijaro J.R., Verhoeven D., Page C.A., Turner D., Farber D.L. (2010). Memory CD4 T cells direct protective responses to influenza virus in the lungs through helper-independent mechanisms. J. Virol..

[45] Brown D.M., Lee S., Garcia-Hernandez Mde L., Swain S.L. (2012). Multifunctional CD4 cells expressing gamma interferon and perforin mediate protection against lethal influenza virus infection. J. Virol..

[46] Bot A., Bot S., Bona C.A. (1998). Protective role of gamma interferon during the recall response to influenza virus. J. Virol..

[47] Krueger P.D., Goldberg M.F., Hong S.W., Osum K.C., Langlois R.A., Kotov D.I., Dileepan T., Jenkins M.K. (2021). Two sequential activation modules control the differentiation of protective T helper-1 (Th1) cells. Immunity.

[48] Califano D., Furuya Y., Roberts S., Avram D., McKenzie A.N.J., Metzger D.W. (2018). IFN-gamma increases susceptibility to influenza A infection through suppression of group II innate lymphoid cells. Mucosal Immunol..

[49] Nicol M.Q., Campbell G.M., Shaw D.J., Dransfield I., Ligertwood Y., Beard P.M., Nash A.A., Dutia B.M. (2019). Lack of IFNgamma signaling attenuates spread of influenza A virus in vivo and leads to reduced pathogenesis. Virology.

[50] Liu B., Bao L., Wang L., Li F., Wen M., Li H., Deng W., Zhang X., Cao B. (2019). Anti-IFN-gamma therapy alleviates acute lung injury induced by severe influenza A (H1N1) pdm09 infection in mice. J. Microbiol. Immunol. Infect..

[51] Schmit T., Guo K., Tripathi J.K., Wang Z., McGregor B., Klomp M., Ambigapathy G., Mathur R., Hur J., Pichichero M. (2022). Interferon-gamma promotes monocyte-mediated lung injury during influenza infection. Cell Rep..

[52] Strutt T.M., McKinstry K.K., Marshall N.B., Vong A.M., Dutton R.W., Swain S.L. (2013). Multipronged CD4^+^ T-cell effector and memory responses cooperate to provide potent immunity against respiratory virus. Immunol. Rev..

[53] Marshall H.D., Chandele A., Jung Y.W., Meng H., Poholek A.C., Parish I.A., Rutishauser R., Cui W., Kleinstein S.H., Craft J. (2011). Differential expression of Ly6C and T-bet distinguish effector and memory Th1 CD4(+) cell properties during viral infection. Immunity.

[54] Wu C.Y., Kirman J.R., Rotte M.J., Davey D.F., Perfetto S.P., Rhee E.G., Freidag B.L., Hill B.J., Douek D.C., Seder R.A. (2002). Distinct lineages of T(H)1 cells have differential capacities for memory cell generation in vivo. Nat. Immunol..

[55] McKinstry K.K., Strutt T.M., Bautista B., Zhang W., Kuang Y., Cooper A.M., Swain S.L. (2014). Effector CD4 T-cell transition to memory requires late cognate interactions that induce autocrine IL-2. Nat. Commun..

[56] Zens K.D., Chen J.K., Guyer R.S., Wu F.L., Cvetkovski F., Miron M., Farber D.L. (2017). Reduced generation of lung tissue-resident memory T cells during infancy. J. Exp. Med..

[57] Hamada H., Garcia-Hernandez Mde L., Reome J.B., Misra S.K., Strutt T.M., McKinstry K.K., Cooper A.M., Swain S.L., Dutton R.W. (2009). Tc17, a unique subset of CD8 T cells that can protect against lethal influenza challenge. J. Immunol..

[58] McKinstry K.K., Strutt T.M., Buck A., Curtis J.D., Dibble J.P., Huston G., Tighe M., Hamada H., Sell S., Dutton R.W. (2009). IL-10 deficiency unleashes an influenza-specific Th17 response and enhances survival against high-dose challenge. J. Immunol..

[59] Maroof A., Yorgensen Y.M., Li Y., Evans J.T. (2014). Intranasal vaccination promotes detrimental Th17-mediated immunity against influenza infection. PLoS Pathog..

[60] Crowe C.R., Chen K., Pociask D.A., Alcorn J.F., Krivich C., Enelow R.I., Ross T.M., Witztum J.L., Kolls J.K. (2009). Critical role of IL-17RA in immunopathology of influenza infection. J. Immunol..

[61] Gopal R., Rangel-Moreno J., Fallert Junecko B.A., Mallon D.J., Chen K., Pociask D.A., Connell T.D., Reinhart T.A., Alcorn J.F., Ross T.M. (2014). Mucosal pre-exposure to Th17-inducing adjuvants exacerbates pathology after influenza infection. Am. J. Pathol..

[62] Liu X., Nguyen T.H., Sokulsky L., Li X., Garcia Netto K., Hsu A.C., Liu C., Laurie K., Barr I., Tay H. (2021). IL-17A is a common and critical driver of impaired lung function and immunopathology induced by influenza virus, rhinovirus and respiratory syncytial virus. Respirology.

[63] Li C., Yang P., Sun Y., Li T., Wang C., Wang Z., Zou Z., Yan Y., Wang W., Wang C. (2012). IL-17 response mediates acute lung injury induced by the 2009 pandemic influenza A (H1N1) virus. Cell Res.

[64] Navaeiseddighi Z., Tripathi J.K., Guo K., Wang Z., Schmit T., Brooks D.R., Allen R.A., Hur J., Mathur R., Jurivich D. (2023). IL-17RA promotes pathologic epithelial inflammation in a mouse model of upper respiratory influenza infection. PLoS Pathog..

[65] Wang X., Chan C.C., Yang M., Deng J., Poon V.K., Leung V.H., Ko K.H., Zhou J., Yuen K.Y., Zheng B.J. (2011). A critical role of IL-17 in modulating the B-cell response during H5N1 influenza virus infection. Cell Mol. Immunol..

[66] Wang X., Ma K., Chen M., Ko K.H., Zheng B.J., Lu L. (2016). IL-17A Promotes Pulmonary B-1a Cell Differentiation via Induction of Blimp-1 Expression during Influenza Virus Infection. PLoS Pathog..

[67] Eliasson D.G., Omokanye A., Schon K., Wenzel U.A., Bernasconi V., Bemark M., Kolpe A., El Bakkouri K., Ysenbaert T., Deng L. (2018). M2e-tetramer-specific memory CD4 T cells are broadly protective against influenza infection. Mucosal Immunol..

[68] Omokanye A., Ong L.C., Lebrero-Fernandez C., Bernasconi V., Schon K., Stromberg A., Bemark M., Saelens X., Czarnewski P., Lycke N. (2022). Clonotypic analysis of protective influenza M2e-specific lung resident Th17 memory cells reveals extensive functional diversity. Mucosal Immunol..

[69] Hornick E.E., Zacharias Z.R., Legge K.L. (2019). Kinetics and Phenotype of the CD4 T Cell Response to Influenza Virus Infections. Front. Immunol..

[70] Muranski P., Restifo N.P. (2013). Essentials of Th17 cell commitment and plasticity. Blood.

[71] Dhume K., Finn C.M., Devarajan P., Singh A., Tejero J.D., Prokop E., Strutt T.M., Sell S., Swain S.L., McKinstry K.K. (2022). Bona Fide Th17 Cells without Th1 Functional Plasticity Protect against Influenza. J. Immunol..

[72] Alcorn J.F. (2020). IL-22 Plays a Critical Role in Maintaining Epithelial Integrity During Pulmonary Infection. Front. Immunol..

[73] Dienz O., Eaton S.M., Bond J.P., Neveu W., Moquin D., Noubade R., Briso E.M., Charland C., Leonard W.J., Ciliberto G. (2009). The induction of antibody production by IL-6 is indirectly mediated by IL-21 produced by CD4^+^ T cells. J. Exp. Med..

[74] Liang S., Mozdzanowska K., Palladino G., Gerhard W. (1994). Heterosubtypic immunity to influenza type A virus in mice. Effector mechanisms and their longevity. J. Immunol..

[75] Intlekofer A.M., Banerjee A., Takemoto N., Gordon S.M., Dejong C.S., Shin H., Hunter C.A., Wherry E.J., Lindsten T., Reiner S.L. (2008). Anomalous type 17 response to viral infection by CD8^+^ T cells lacking T-bet and eomesodermin. Science.

[76] Paiva I.A., Badolato-Correa J., Familiar-Macedo D., de-Oliveira-Pinto L.M. (2021). Th17 Cells in Viral Infections-Friend or Foe?. Cells.

[77] Aghbash P.S., Hemmat N., Nahand J.S., Shamekh A., Memar M.Y., Babaei A., Baghi H.B. (2021). The role of Th17 cells in viral infections. Int. Immunopharmacol..

[78] Paroli M., Caccavale R., Fiorillo M.T., Spadea L., Gumina S., Candela V., Paroli M.P. (2022). The Double Game Played by Th17 Cells in Infection: Host Defense and Immunopathology. Pathogens.

[79] McKinstry K.K., Strutt T.M., Swain S.L. (2010). Regulation of CD4^+^ T-cell contraction during pathogen challenge. Immunol. Rev..

[80] Gao X., Luo K., Wang D., Wei Y., Yao Y., Deng J., Yang Y., Zeng Q., Dong X., Xiong L. (2023). T follicular helper 17 (Tfh17) cells are superior for immunological memory maintenance. Elife.

[81] Rynda-Apple A., Robinson K.M., Alcorn J.F. (2015). Influenza and Bacterial Superinfection: Illuminating the Immunologic Mechanisms of Disease. Infect. Immun..

[82] McCullers J.A. (2014). The co-pathogenesis of influenza viruses with bacteria in the lung. Nat. Rev. Microbiol..

[83] George S.T., Lai J., Ma J., Stacey H.D., Miller M.S., Mullarkey C.E. (2021). Neutrophils and Influenza: A Thin Line between Helpful and Harmful. Vaccines.

[84] Paget C., Trottein F. (2019). Mechanisms of Bacterial Superinfection Post-influenza: A Role for Unconventional T Cells. Front. Immunol..

[85] Er J.Z., Koean R.A.G., Ding J.L. (2019). Loss of T-bet confers survival advantage to influenza-bacterial superinfection. EMBO J..

[86] Koutsakos M., McWilliam H.E.G., Aktepe T.E., Fritzlar S., Illing P.T., Mifsud N.A., Purcell A.W., Rockman S., Reading P.C., Vivian J.P. (2019). Downregulation of MHC Class I Expression by Influenza A and B Viruses. Front. Immunol..

[87] Devarajan P., Vong A.M., Castonguay C.H., Silverstein N.J., Kugler-Umana O., Bautista B.L., Kelly K.A., Luban J., Swain S.L. (2023). Cytotoxic CD4 development requires CD4 effectors to concurrently recognize local antigen and encounter type I IFN-induced IL-15. Cell Rep..

[88] Brown D.M., Kamperschroer C., Dilzer A.M., Roberts D.M., Swain S.L. (2009). IL-2 and antigen dose differentially regulate perforin- and FasL-mediated cytolytic activity in antigen specific CD4+ T cells. Cell Immunol..

[89] Esser M.T., Dinglasan R.D., Krishnamurthy B., Gullo C.A., Graham M.B., Braciale V.L. (1997). IL-2 induces Fas ligand/Fas (CD95L/CD95) cytotoxicity in CD8+ and CD4+ T lymphocyte clones. J. Immunol..

[90] Read K.A., Jones D.M., Pokhrel S., Hales E.D.S., Varkey A., Tuazon J.A., Eisele C.D., Abdouni O., Saadey A., Leonard M.R. (2023). Aiolos represses CD4^+^ T cell cytotoxic programming via reciprocal regulation of T(FH) transcription factors and IL-2 sensitivity. Nat. Commun..

[91] Preglej T., Ellmeier W. (2022). CD4(+) Cytotoxic T cells-Phenotype, Function and Transcriptional Networks Controlling Their Differentiation Pathways. Immunol. Lett..

[92] Hua L., Yao S., Pham D., Jiang L., Wright J., Sawant D., Dent A.L., Braciale T.J., Kaplan M.H., Sun J. (2013). Cytokine-dependent induction of CD4^+^ T cells with cytotoxic potential during influenza virus infection. J. Virol..

[93] Marshall N.B., Vong A.M., Devarajan P., Brauner M.D., Kuang Y., Nayar R., Schutten E.A., Castonguay C.H., Berg L.J., Nutt S.L. (2017). NKG2C/E Marks the Unique Cytotoxic CD4 T Cell Subset, ThCTL, Generated by Influenza Infection. J. Immunol..

[94] Kamperschroer C., Swain S.L., Grussenmeyer T., Lefkovits I. (2006). SAP deficiency results in a striking alteration of the protein profile in activated CD4 T cells. J. Proteome Res..

[95] Cannons J.L., Yu L.J., Jankovic D., Crotty S., Horai R., Kirby M., Anderson S., Cheever A.W., Sher A., Schwartzberg P.L. (2006). SAP regulates T cell-mediated help for humoral immunity by a mechanism distinct from cytokine regulation. J. Exp. Med..

[96] Karnowski A., Chevrier S., Belz G.T., Mount A., Emslie D., D’Costa K., Tarlinton D.M., Kallies A., Corcoran L.M. (2012). B and T cells collaborate in antiviral responses via IL-6, IL-21, and transcriptional activator and coactivator, Oct2 and OBF-1. J. Exp. Med..

[97] Ballesteros-Tato A., Leon B., Graf B.A., Moquin A., Adams P.S., Lund F.E., Randall T.D. (2012). Interleukin-2 inhibits germinal center formation by limiting T follicular helper cell differentiation. Immunity.

[98] Schultz-Cherry S., Hinshaw V.S. (1996). Influenza virus neuraminidase activates latent transforming growth factor beta. J. Virol..

[99] Marshall H.D., Ray J.P., Laidlaw B.J., Zhang N., Gawande D., Staron M.M., Craft J., Kaech S.M. (2015). The transforming growth factor beta signaling pathway is critical for the formation of CD4 T follicular helper cells and isotype-switched antibody responses in the lung mucosa. Elife.

[100] Yoo J.K., Fish E.N., Braciale T.J. (2012). LAPCs promote follicular helper T cell differentiation of Ag-primed CD4^+^ T cells during respiratory virus infection. J. Exp. Med..

[101] Devarajan P., Vong A.M., Castonguay C.H., Kugler-Umana O., Bautista B.L., Jones M.C., Kelly K.A., Xia J., Swain S.L. (2022). Strong influenza-induced T(FH) generation requires CD4 effectors to recognize antigen locally and receive signals from continuing infection. Proc. Natl. Acad. Sci. USA.

[102] Krishnaswamy J.K., Gowthaman U., Zhang B., Mattsson J., Szeponik L., Liu D., Wu R., White T., Calabro S., Xu L. (2017). Migratory CD11b(+) conventional dendritic cells induce T follicular helper cell-dependent antibody responses. Sci. Immunol..

[103] Horiuchi S., Wu H., Liu W.C., Schmitt N., Provot J., Liu Y., Bentebibel S.E., Albrecht R.A., Schotsaert M., Forst C.V. (2021). Tox2 is required for the maintenance of GC T(FH) cells and the generation of memory T(FH) cells. Sci. Adv..

[104] Miyauchi K., Adachi Y., Tonouchi K., Yajima T., Harada Y., Fukuyama H., Deno S., Iwakura Y., Yoshimura A., Hasegawa H. (2021). Influenza virus infection expands the breadth of antibody responses through IL-4 signalling in B cells. Nat. Commun..

[105] Miyauchi K., Sugimoto-Ishige A., Harada Y., Adachi Y., Usami Y., Kaji T., Inoue K., Hasegawa H., Watanabe T., Hijikata A. (2016). Protective neutralizing influenza antibody response in the absence of T follicular helper cells. Nat. Immunol..

[106] Bentebibel S.E., Lopez S., Obermoser G., Schmitt N., Mueller C., Harrod C., Flano E., Mejias A., Albrecht R.A., Blankenship D. (2013). Induction of ICOS+CXCR3+CXCR5+ TH cells correlates with antibody responses to influenza vaccination. Sci. Transl. Med..

[107] Spensieri F., Borgogni E., Zedda L., Bardelli M., Buricchi F., Volpini G., Fragapane E., Tavarini S., Finco O., Rappuoli R. (2013). Human circulating influenza-CD4^+^ ICOS1^+^IL-21^+^ T cells expand after vaccination, exert helper function, and predict antibody responses. Proc. Natl. Acad. Sci. USA.

[108] He J., Tsai L.M., Leong Y.A., Hu X., Ma C.S., Chevalier N., Sun X., Vandenberg K., Rockman S., Ding Y. (2013). Circulating precursor CCR7(lo)PD-1(hi) CXCR5(+) CD4(+) T cells indicate Tfh cell activity and promote antibody responses upon antigen reexposure. Immunity.

[109] Son Y.M., Cheon I.S., Wu Y., Li C., Wang Z., Gao X., Chen Y., Takahashi Y., Fu Y.X., Dent A.L. (2021). Tissue-resident CD4^+^ T helper cells assist the development of protective respiratory B and CD8^+^ T cell memory responses. Sci. Immunol..

[110] Cullen J.G., McQuilten H.A., Quinn K.M., Olshansky M., Russ B.E., Morey A., Wei S., Prier J.E., La Gruta N.L., Doherty P.C. (2019). CD4^+^ T help promotes influenza virus-specific CD8^+^ T cell memory by limiting metabolic dysfunction. Proc. Natl. Acad. Sci. USA.

[111] Laidlaw B.J., Zhang N., Marshall H.D., Staron M.M., Guan T., Hu Y., Cauley L.S., Craft J., Kaech S.M. (2014). CD4+ T cell help guides formation of CD103+ lung-resident memory CD8+ T cells during influenza viral infection. Immunity.

[112] Lee B.O., Hartson L., Randall T.D. (2003). CD40-deficient, influenza-specific CD8 memory T cells develop and function normally in a CD40-sufficient environment. J. Exp. Med..

[113] Olson M.R., Seah S.G., Cullen J., Greyer M., Edenborough K., Doherty P.C., Bedoui S., Lew A.M., Turner S.J. (2014). Helping themselves: Optimal virus-specific CD4 T cell responses require help via CD4 T cell licensing of dendritic cells. J. Immunol..

[114] Zloza A., Kohlhapp F.J., Lyons G.E., Schenkel J.M., Moore T.V., Lacek A.T., O’Sullivan J.A., Varanasi V., Williams J.W., Jagoda M.C. (2012). NKG2D signaling on CD8^+^ T cells represses T-bet and rescues CD4-unhelped CD8^+^ T cell memory recall but not effector responses. Nat. Med..

[115] Betts R.J., Prabhu N., Ho A.W., Lew F.C., Hutchinson P.E., Rotzschke O., Macary P.A., Kemeny D.M. (2012). Influenza A virus infection results in a robust, antigen-responsive, and widely disseminated Foxp3+ regulatory T cell response. J. Virol..

[116] Bedoya F., Cheng G.S., Leibow A., Zakhary N., Weissler K., Garcia V., Aitken M., Kropf E., Garlick D.S., Wherry E.J. (2013). Viral antigen induces differentiation of Foxp3+ natural regulatory T cells in influenza virus-infected mice. J. Immunol..

[117] Antunes I., Kassiotis G. (2010). Suppression of innate immune pathology by regulatory T cells during Influenza A virus infection of immunodeficient mice. J. Virol..

[118] Griffith J.W., Faustino L.D., Cottrell V.I., Nepal K., Hariri L.P., Chiu R.S., Jones M.C., Jule A., Gabay C., Luster A.D. (2023). Regulatory T cell-derived IL-1Ra suppresses the innate response to respiratory viral infection. Nat. Immunol..

[119] Brincks E.L., Roberts A.D., Cookenham T., Sell S., Kohlmeier J.E., Blackman M.A., Woodland D.L. (2013). Antigen-specific memory regulatory CD4+Foxp3+ T cells control memory responses to influenza virus infection. J. Immunol..

[120] Lu C., Chen W. (2021). Influenza virus infection selectively triggers the accumulation and persistence of more potent Helios-expressing Foxp3(+) regulatory T cells in the lungs. Immunol. Cell Biol..

[121] Kaiser K.A., Loffredo L.F., Santos-Alexis K.L., Ringham O.R., Arpaia N. (2023). Regulation of the alveolar regenerative niche by amphiregulin-producing regulatory T cells. J. Exp. Med..

[122] Leon B., Bradley J.E., Lund F.E., Randall T.D., Ballesteros-Tato A. (2014). FoxP3+ regulatory T cells promote influenza-specific Tfh responses by controlling IL-2 availability. Nat. Commun..

[123] Lu Y., Jiang R., Freyn A.W., Wang J., Strohmeier S., Lederer K., Locci M., Zhao H., Angeletti D., O’Connor K.C. (2021). CD4^+^ follicular regulatory T cells optimize the influenza virus-specific B cell response. J. Exp. Med..

[124] Abbott C.A., Freimayer E.L., Tyllis T.S., Norton T.S., Alsharifi M., Heng A.H.S., Pederson S.M., Qu Z., Armstrong M., Hill G.R. (2023). Determination of Tr1 cell populations correlating with distinct activation states in acute IAV infection. Mucosal Immunol..

[125] Sun K., Torres L., Metzger D.W. (2010). A detrimental effect of interleukin-10 on protective pulmonary humoral immunity during primary influenza A virus infection. J. Virol..

[126] Sun J., Madan R., Karp C.L., Braciale T.J. (2009). Effector T cells control lung inflammation during acute influenza virus infection by producing IL-10. Nat. Med..

[127] Gu Y., Yang J., Ouyang X., Liu W., Li H., Yang J., Bromberg J., Chen S.H., Mayer L., Unkeless J.C. (2008). Interleukin 10 suppresses Th17 cytokines secreted by macrophages and T cells. Eur. J. Immunol..

[128] Dutta A., Huang C.T., Chen T.C., Lin C.Y., Chiu C.H., Lin Y.C., Chang C.S., He Y.C. (2015). IL-10 inhibits neuraminidase-activated TGF-beta and facilitates Th1 phenotype during early phase of infection. Nat. Commun..

[129] Huang W., Solouki S., Koylass N., Zheng S.G., August A. (2017). ITK signalling via the Ras/IRF4 pathway regulates the development and function of Tr1 cells. Nat. Commun..

[130] Subrahmanyam P.B., Holmes T.H., Lin D., Su L.F., Obermoser G., Banchereau J., Pascual V., Garcia-Sastre A., Albrecht R.A., Palucka K. (2020). Mass Cytometry Defines Virus-Specific CD4(+) T Cells in Influenza Vaccination. Immunohorizons.

[131] Marinaik C.B., Kingstad-Bakke B., Lee W., Hatta M., Sonsalla M., Larsen A., Neldner B., Gasper D.J., Kedl R.M., Kawaoka Y. (2020). Programming Multifaceted Pulmonary T Cell Immunity by Combination Adjuvants. Cell Rep. Med..

[132] Jang Y.H., Seong B.L. (2021). Immune Responses Elicited by Live Attenuated Influenza Vaccines as Correlates of Universal Protection against Influenza Viruses. Vaccines.

[133] Zheng M.Z.M., Wakim L.M. (2022). Tissue resident memory T cells in the respiratory tract. Mucosal Immunol..

[134] Zens K.D., Chen J.K., Farber D.L. (2016). Vaccine-generated lung tissue-resident memory T cells provide heterosubtypic protection to influenza infection. JCI Insight.

[135] Strutt T.M., Dhume K., Finn C.M., Hwang J.H., Castonguay C., Swain S.L., McKinstry K.K. (2018). IL-15 supports the generation of protective lung-resident memory CD4 T cells. Mucosal Immunol..

[136] Strutt T.M., McKinstry K.K., Dibble J.P., Winchell C., Kuang Y., Curtis J.D., Huston G., Dutton R.W., Swain S.L. (2010). Memory CD4^+^ T cells induce innate responses independently of pathogen. Nat. Med..

[137] Chapman T.J., Lambert K., Topham D.J. (2011). Rapid reactivation of extralymphoid CD4 T cells during secondary infection. PLoS ONE.

[138] Blanco-Lobo P., Nogales A., Rodriguez L., Martinez-Sobrido L. (2019). Novel Approaches for The Development of Live Attenuated Influenza Vaccines. Viruses.

[139] Ullah S., Ross T.M. (2022). Next generation live-attenuated influenza vaccine platforms. Expert. Rev. Vaccines.

[140] Bautista B.L., Devarajan P., McKinstry K.K., Strutt T.M., Vong A.M., Jones M.C., Kuang Y., Mott D., Swain S.L. (2016). Short-Lived Antigen Recognition but Not Viral Infection at a Defined Checkpoint Programs Effector CD4 T Cells To Become Protective Memory. J. Immunol..

[141] Swain S.L., Jones M.C., Devarajan P., Xia J., Dutton R.W., Strutt T.M., McKinstry K.K. (2021). Durable CD4 T-Cell Memory Generation Depends on Persistence of High Levels of Infection at an Effector Checkpoint that Determines Multiple Fates. Cold Spring Harb. Perspect. Biol..

[142] McKinstry K.K., Alam F., Flores-Malavet V., Nagy M.Z., Sell S., Cooper A.M., Swain S.L., Strutt T.M. (2019). Memory CD4 T cell-derived IL-2 synergizes with viral infection to exacerbate lung inflammation. PLoS Pathog..

[143] Alam F., Singh A., Flores-Malavet V., Sell S., Cooper A.M., Swain S.L., McKinstry K.K., Strutt T.M. (2020). CD25-Targeted IL-2 Signals Promote Improved Outcomes of Influenza Infection and Boost Memory CD4 T Cell Formation. J. Immunol..

[144] Stephens R., Langhorne J. (2006). Priming of CD4^+^ T cells and development of CD4^+^ T cell memory; lessons for malaria. Parasite Immunol..

[145] Sant A.J., DiPiazza A.T., Nayak J.L., Rattan A., Richards K.A. (2018). CD4 T cells in protection from influenza virus: Viral antigen specificity and functional potential. Immunol. Rev..

[146] Moritzky S.A., Richards K.A., Glover M.A., Krammer F., Chaves F.A., Topham D.J., Branche A., Nayak J.L., Sant A.J. (2023). The Negative Effect of Preexisting Immunity on Influenza Vaccine Responses Transcends the Impact of Vaccine Formulation Type and Vaccination History. J. Infect. Dis..

[147] Brickley E.B., Wright P.F., Khalenkov A., Neuzil K.M., Ortiz J.R., Rudenko L., Levine M.Z., Katz J.M., Brooks W.A. (2019). The Effect of Preexisting Immunity on Virus Detection and Immune Responses in a Phase II, Randomized Trial of a Russian-Backbone, Live, Attenuated Influenza Vaccine in Bangladeshi Children. Clin. Infect. Dis..

[148] Ng T., Flores-Malavet V., Mansoor M.A.M., Arvelo A.C., Dhume K., Prokop E., McKinstry K.K., Strutt T.M. (2023). Intermediate Levels of Pre-Existing Protective Antibody Allow Priming of Protective T Cell Immunity against Influenza. J. Immunol..

[149] Lobby J.L., Danzy S., Holmes K.E., Lowen A.C., Kohlmeier J.E. (2024). Both Humoral and Cellular Immunity Limit the Ability of Live Attenuated Influenza Vaccines to Promote T Cell Responses. J. Immunol..

[150] Ward B.J., Pillet S., Charland N., Trepanier S., Couillard J., Landry N. (2018). The establishment of surrogates and correlates of protection: Useful tools for the licensure of effective influenza vaccines?. Hum. Vaccin. Immunother..

[151] Westerhof L.M., Noonan J., Hargrave K.E., Chimbayo E.T., Cheng Z., Purnell T., Jackson M.R., Borcherding N., MacLeod M.K.L. (2023). Multifunctional cytokine production marks influenza A virus-specific CD4 T cells with high expression of survival molecules. Eur. J. Immunol..

